# Use of Risk Assessment Tool for Inpatient Traumatic Intracranial Hemorrhage after Falls in Acute Care Hospital Setting

**DOI:** 10.5539/gjhs.v4n3p64

**Published:** 2012-05-01

**Authors:** Shin-ichi Toyabe

**Affiliations:** 1 Niigata University Crisis Mangement Office, Niigata University Hospital, Niigata City, Japan

**Keywords:** falls, STRATIFY, FRAX, traumatic intracranial hemorrhage, traumatic bone fracture

## Abstract

Severe injuries such as intracranial hemorrhage (ICH) are the most serious problem after falls in hospital, but they have not been considered in risk assessment scores for falls. We tried to determine the risk factors for ICH after falls in 20,320 inpatients (696,364 patient-days) aged from 40 to 90 years who were admitted to a tertiary-care university hospital. Possible risk factors including STRATIFY risk score for falls and FRAX™ risk score for fractures were analyzed by univariate and multivariate analyses. Fallers accounted for 3.2% of the patients, and 5.0% of the fallers suffered major injuries, including peripheral bone fracture (59.6%) and ICH (23.4%). In addition to STRATIFY, FRAX™ was significantly associated not only with bone fractures but also ICH. Concomitant use of risk score for falls and risk score for fractures might be useful for the prediction of major injuries such as ICH after falls.

## 1. Introduction

Falls are very common accidents in hospitals (Nakai et al, 2006). Falls in a hospital often cause severe injuries such as bone fractures, soft tissue injuries and hematomas. About 3-10% of falls in hospitals result in these physical injuries (Fischer et al, 2005). These injuries may lead to additional healthcare costs, prolonged length of stay and psychological distress for patients, which might result in complaints and litigation from families of the patients (Oliver et al, 2006). One strategy to prevent these situations is targeted intervention for patients who are judged to be at high risk of falls by risk assessment tools for falls (Gates et al, 2008; Rubenstein, 2006; Haines et al, 2007). However, these risk assessment tools were developed to identify patients at high risk for falls, not to predict patients who would suffer physical injuries after falls. The most important reason for preventing falls should be to prevent severe injuries. Risk assessment tools are needed to predict falls that are likely to be complicated with severe injuries. We have recently reported that risk assessment tools for osteoporotic fractures might be useful for prediction of fractures after falls (Toyabe, 2010).

Among the various injuries that can occur after falls, intracranial hemorrhage (ICH) is one of the most serious. Falls are a leading cause of traumatic ICH in emergency department visits (Gangavati et al, 2009; Thompson et al, 2006), and patients at high risk for falls have been reported to be at increased risk for ICH (Gage, 2005). Falls are an independent risk factor for ICH in patients with atrial fibrillation. An increased number of patients is now taking anticoagulant drugs and antiplatelet drugs for various conditions such as atrial fibrillation, valvular heart diseases and thrombotic diseases (Gangavati et al, 2009). Since ICH is the most serious complication of administration of these drugs, patients who are given these drugs are thought to be more prone to ICH after falls. However, again, risk assessment tools for falls were not developed to identify patients who would suffer ICH after falls.

The aims of this study were (i) to determine the prevalence of ICH after falls and (ii) to elucidate the risk factors for ICH after falls among various patient characteristics.

## 2. Methods

### 2.1 Settings

This study was conducted at Niigata University Hospital, an 810-bed academic teaching hospital in the city of Niigata. There are 23 clinical departments and the service area of the hospital as a tertiary care hospital covers all districts in Niigata Prefecture, which has a population of 2,400,000. All patients who had been admitted to the hospital during the period from April 2006 to March 2010 and who were aged from 40 to 90 years at admission were studied. The reason why we selected subjects aged from 40 to 90 years is that the FRAX™ scoring system to assess patients’ risk for fractures targets that age group (Fujiwara et al, 2008; Kanis et al, 2005; WHO, 2008). During that period, 29,110 patients (696,365 patient-days) aged from 40 to 90 years (median, 65.0 years; 25th percentile, 56.0 years; 75th percentile, 74.0 years) were admitted in our hospital. The patients included 13,792 males and 15,318 females. Acute admission was required in 7,160 (24.6%) of the patients.

### 2.2 Risk Assessment Scores for Falls and Fractures

The STRATIFY risk assessment score (St. Thomas risk assessment tool in falling elderly) was used to assess patients’ risk for falls in our study (Oliver et al, 1997). The STRATIFY score is based on five factors (with or without history of falls, agitated confusion, visual impairment, urinary frequency and high transfer/mobility score), and a score of more than two was considered as high risk for falls (Toyabe, 2010). The FRAX™ score was proposed by WHO to compute ten-year probability of osteoporotic fracture by integrating various risk factors for osteoporosis. These risk factors include age, prior fragility fracture, parental history of hip fracture, smoking, use of systemic corticosteroids, excess alcohol intake and rheumatoid arthritis. The FRAX™ score has the advantages that it can be used without information on bone mineral density and that it is adjusted for ethnic differences. In this study, we used the FRAX™ score calculated on the basis of the ten-year probability of major osteoporotic fracture in Japanese population.

### 2.3 Data Collection

Information on patients’ background such as age, gender, body weight, height, history of bone fractures, smoking history, alcoholic consumption, prescription of drugs, coexisting illness, admission day and discharge day was obtained from the hospital information system. Among the drugs prescribed for the patients, we focused on ‘culprit’ drugs for falls and drugs that may cause hemorrhagic tendency such as antiplatelets and anticoagulants. ‘Culprit’ drugs for falls include centrally sedating agents (sedatives, hypnotics, opiates and anticonvulsants) and drugs that can precipitate postural hypotension, arrhythmia or syncope (antihypertensives, diuretics and antiarrhythmics). Laboratory findings related to hemorrhagic tendency and platelet counts were not included as possible risk factors for ICH in this study because these tests were not performed in all patients. Information on risk factors for falls was obtained from medical charts of the patients and fall assessment records completed by the attending nurse at admission. The assessment was performed again when fall events occurred. Background diseases of the patients were classified according to ICD-10 codings, which consist of 12 main classes.

### 2.4 Falls and Major Injuries after Falls

The clinical outcome we studied was fallers with or without major injuries rather than falls (Papaioannou et al, 2004; Lamb et al, 2005). Data on inpatient fall events were obtained from patient safety incident reports, records of radiographic examination and medical charts of the patients. The patient safety incidents are reported voluntarily through intra-institutional online reporting system. The incident reports concerning fall events were documented by attending nurses and other medical staff, and the reports contained data on time, location, injuries sustained and potential causative factors for falls. Major injuries after falls correspond to cases in which the degree of harm is moderate, severe or fatal in terms of the framework of the international classification of patient safety (WHO, 2009). Therefore, major injuries include bone fractures, dislocations of the joints, intracranial hemorrhages, excessive serious bleedings or lacerations and multiple injuries. Pains, bruises, isolated hematomas and superficial wounds were excluded from major injuries. As for fractures, only cases with bone fracture verified by radiographic examination were included. Vertebral compression fractures were included when they were not found in radiographic examinations before the events but were first detected by radiographic examinations after falls. Diagnosis of intracranial hemorrhage and its location and extent was determined by computed tomography.

### 2.5 Statistical Analyses

Fall events and major injuries after falls were analyzed by two different methods. The first method is the traditional chi-square test and multiple logistic analysis, which has been used for analysis of inpatient falls. For multiple logistic analysis, significant risk factors were selected by using the stepwise selection method. The second method is survival analysis in which time between admission and the event (falls or major injuries after falls) during the hospital stay was considered as survival time. Discharge of the patient without fall events was considered as censoring. The reason why we used survival analyses is that length of stay in acute care hospitals in Japan is very long as compared with other countries (OECD, 2010). The Kaplan-Meier method was used for analysis, and the logrank test was used to examine whether each risk factor was significantly associated with events. A multivariate Cox’s regression model was used to examine the risk factors that were most significantly associated with events among the various risk factors. Significant factors were selected by the stepwise selection method. A cut-off value to distinguish patients at risk from patients not at a risk was determined on the basis of results of receiver-operating curve (ROC) analysis and Youden index. Sensitivity, specificity and area under the ROC (AUC) were calculated to examine the performance of the cut-off values to detect events. The association between two continuous variables was analyzed by Spearman rank order coefficient. Distribution of continuous data was shown by medians (25-percentile, 75-percentile). All statistical analyses were performed using IBM SPSS Statistics 19 (SPSS Japan Inc., Tokyo, Japan), and a p-value less than 0.05 was considered significant.

### 2.6 Ethical Consideration

All data were analyzed anonymously. The Ethics Committee of Niigata University School of Medicine gave ethical approval for the study.

## 3. Results

### 3.1 Falls and Major Injuries after Falls

Of the 29,110 inpatients, 933 (3.2%) experienced more than one fall during admission. Forty-seven (5.0%) of the fallers suffered major injuries. The most frequent major injury was peripheral bone fracture (28 cases, 59.6%) and the second most frequent major injury was ICH (11 cases, 23.4%). The third most frequent major injury was vertebral compression fracture, which was first detected by radiographic examination after a fall (5 cases, 10.6%). Other cases included disruption of surgical wounds, brain contusion and rupture of a liver tumor.

### 3.2 Oral Anticoagulants and Antiplatelets

Various oral anticoagulants and/or antiplatelets were given to 16.7% of the patients. Warfarin and aspirin were the most commonly administered drugs. Warfarin was administered to 9.0% of the patients and aspirin was administered to 7.8% of the patients. Other drugs given to the patients included trapidil, ticlopidine hydrochloride, dilazepdihydrochloride, dipyridamole, cilostazol, ifenprodil tartrate, limaprostalfadex, ibudilast, salpogrelate hydrochloride and beraprost sodium.

### 3.3 Characteristics of the Patients with ICH after Falls

**Figure 1:**
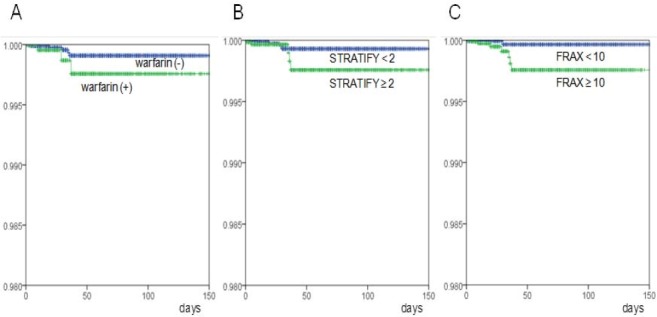
Incidences of ICH after falls were compared for patients who were taking warfarin and patients who were not (A), patients with STRATIFY score of more than 2 and patients with STRATIFY score of less than 2 (B), and patients with FRAX™ score of more than 10 and patients with FRAX™ score of less than 10 (C). Horizontal bar indicates length of hospital stay (days) before ICH developed

Localizations of the ICH in the 11 patients were epidural (2 patients), subdural (4), subarachnoid (4) and intracerebral (1). The patient with intracerebral hemorrhage suffered frontal bone fracture. Six (54.4%) of the patients were given anticoagulants and/or antiplatelets. Hemorrhagic tendency due to underlying diseases or administration of anticoagulants and/or antiplatelets was observed in several patients. Among the patients, one patient showed thrombocytopenia of less than 100 x 10^9^/L, and another patient showed prolongation of a PTT to 1.5 times the normal value. Four patients were anticoagulated with warfarin, but only one of the patients showed elevated PT-INR of more than 2.0. One patient was receiving aspirin. The STRATIFY score of the 11 patients with ICH ranged from 0 to 4, and five (45.5%) of the 11 patients had a STRATIFY score of less than 2.

### 3.4 Univariate Analyses of Factors associated with ICH after Falls

Agitated confusion, STRATIFY score, FRAX™ score and administration of anticoagulants and/or antiplatelets were found to be factors significantly associated with ICH after falls (Table 1) in both the chi-square test and logrank test. The ward and the department in which patients were admitted and underlying disease categorized by ICD-10 main classes were significant factors only in the chi-square test. Among various anticoagulants and/or antiplatelets, warfarin was significantly associated with ICH after falls in both tests, but aspirin showed a significant association only in the chi-square test.

**Table 1:** Results of univariate analysis of factors associated with ICH after falls

Items	Number of patients		Sig.	
	
	Fallers with ICH after falls	Non-fallers and fallers without ICH after falls	Logrank test	Chi-square test
n	11	29,099		
Female gender	5	13,787	0.975	1.000
History of falls	4	16,903	0.133	0.220
Gait instability	8	21,752	0.100	0.075
Agitated confusion	6	22,151	<0.001	0.001
Urinary incontinence/frequency	8	21,621	0.108	0.086
Visual impairment	9	18,878	0.991	1.000
Lower limb weakness	6	19,152	0.165	0.052
Prescription of ‘culprit’ drugs	9	20,635	0.924	0.658
STRATIFY score ≥ 2	5	22,710	0.001	0.018
LOS ≥ 14	7	14,147	-	0.376
FRAX™ ≥ 10	8	10,462	0.018	0.008
Ward	-	-	0.162	<0.001
Department	-	-	0.398	<0.001
ICD10	-	-	0.502	0.032
Any anticoagulant and/or antiplatlet drugs	6	4,859	0.027	0.005
Warfarin	4	2,615	0.048	0.013
Aspirin	3	2,276	0.096	0.049

### 3.5 Multivariate Analyses of Factors associated with ICH after Falls

We tried to find factors most closely associated with ICH after falls among the various factors shown in Table 1. Multivariate Cox’s regression analysis showed that STRATIFY score and FRAX™ score were factors most closely associated with ICH after falls (Table 2). By using multiple regression analysis, STRATIFY score, FRAX™ score and use of anticoagulants and/or antiplatelets were found to be significant factors.

**Table 2:** Results of multivariate analyses of factors associated with ICH after falls. Results of multivariate Cox regression analysis (A) and multiple regression analysis are shown. HR, hazard ratio; CI, confidence interval; OR, odds ratio

A.							

Events	variables	Estimated coefficient (β)	Standard error for β	Sig.	HR	95% C.I. for β	
Major injuries	STRATIFY	0.431	0.121	<0.001	1.539	1.214	1.952
	FRAX™	0.048	0.012	<0.001	1.049	1.024	1.074

Peripheral fractures	STRATIFY	0.546	0.154	<0.001	1.726	1.276	2.336
	FRAX™	0.050	0.016	0.001	1.051	1.019	1.083

Fractures	STRATIFY	0.474	0.145	0.001	1.606	1.209	2.133
	FRAX™	0.051	0.014	<0.001	1.052	1.023	1.082

ICH	STRATIFY	0.568	0.233	0.015	1.765	1.119	2.784
	FRAX™	0.053	0.024	0.023	1.055	1.007	1.105

B.							

Dependent variables	Independent variables	Estimated coefficient (β)	Standard error for β	Sig.	OR	95% C.I. for β	
Major injuris	STRATIFY	0.539	0.118	<0.001	1.714	1.359	2.161
	FRAX™	0.052	0.012	<0.001	1.054	1.029	1.079
	constant	-7.780	0.284	<0.001			

Peripheral fractures	STRATIFY	0.625	0.149	<0.001	1.869	1.396	2.502
	FRAX™	0.051	0.016	0.001	1.053	1.021	1.085
constant	-8.444	0.375	<0.001			

Fractures	STRATIFY	0.534	0.141	<0.001	1.706	1.295	2.248
	FRAX™	0.056	0.014	<0.001	1.058	1.029	1.087
constant	-8.191	0.339	<0.001			

ICH	STRATIFY	0.488	0.119	<0.001	1.630	1.290	2.058
	FRAX™	0.050	0.012	<0.001	1.051	1.026	1.077
	Anticoagulants or antiplatelets	1.013	0.301	0.001	2.754	1.527	4.967	
	constant	-7.971	0.299	<0.001			

### 3.6 Performance of the STRATIFY Score and FRAX™ Score

Sensitivity and specificity of the STRATIFY score and FRAX™ score to predict ICH after falls are shown in Table 3A. In all types of events, the FRAX™ score showed superior sensitivity and inferior specificity to the STRATIFY score. ROC analyses were performed to evaluate the performance of these scores (Table 3B). The STRATIFY score showed significantly superior performance to the FRAX™ score for predicting falls. However, the STRATIFY score tended to show poorer performance to detect major injuries including ICH after falls. The Spearman rank order coefficient showed that there was only a slight correlation between the STRATIFY score and FRAX™ score (R=0.207, p<0.001).

**Table 3:** Performance of the STRATIFY score and FRAX™ score. Sensitivity and specificity of the scores to predict ICH were calculated (A), and AUC was calculated (B). AUC values generated by the STRATIFY score and FRAX™ score were compared

A. Sensitivity and specificity			

Events	Scores	Sensitivity	Specificity
Falls	STRATIFY	0.533	0.791
	FRAX™	0.554	0.628
Major injuries after falls	STRATIFY	0.532	0.781
	FRAX™	0.733	0.623
Peripheral fracture after falls	STRATIFY	0.607	0.781
	FRAX™	0.778	0.623
Fracture after falls	STRATIFY	0.545	0.781
	FRAX™	0.781	0.623
ICH after falls	STRATIFY	0.545	0.780
	FRAX™	0.800	0.623

B. Area under the ROC		

Events	STRATIFY	FRAX™	p-value
Fall	0.698	0.615	<0.001
Major injuries after falls	0.683	0.708	0.643
Peripheral fracture after falls	0.718	0.722	0.957
Fracture after falls	0.688	0.720	0.622
ICH after falls	0.713	0.751	0.752

## 3. Discussion

Although the FRAX™ score was originally developed as a tool for predicting osteoporotic fracture (Fujiwara et al, 2008; Kanis et al, 2005; WHO, 2008), we unexpectedly found a significant association between ICH after falls and high FRAX™ score. The association was not due to vulnerability of cranial bone, because only one patient suffered cranial bone fracture after fall among 11 patients with ICH after falls. It is further unlikely that high FRAX™ score is related to hemorrhagic tendency. Rather, high FRAX™ score might be associated with fallers, especially fallers complicated with major injuries such as ICH. Actually, the FRAX™ score was significantly associated with fallers in our study in both the chi-square test and logrank test (data not shown). The performance of the FRAX™ score to detect fallers was inferior than when it was used to detect ICH after falls (Table 3), and only a weak correlation was found between FRAX™ score and STRATIFY score. From these results, the FRAX™ score might detect a group of fall-prone patients who have different characteristics from those who showed high STRATIFY scores. Association between falls and high FRAX™ score gave a new insight into this score, because fall-related risk factors were explicitly excluded from the FRAX™ calculation (van den Bergh et al, 2010).

There have been many reports on the association between osteoporosis and falls. Most of the reports have focused on the relationship between fall-prone patients and vitamin D deficiency (Bischoff-Ferrari et al, 2004). Fall prevention by vitamin D supplementation has been extensively studied (Bischoff-Ferrari et al, 2004 & 2009). A decreased level of serum vitamin D increases body sway and decreases muscle strength, which result in frequent falls (Pfeifer et al, 2002). In addition, high serum parathyroid hormone has been identified as a risk factor for falls independent of vitamin D deficiency (Houston et al, 2008; Sambrook et al, 2004). However, measurements of serum vitamin D and parathyroid hormone are not suitable for screening of fall-prone patients because it is costly and impractical to measure them in all patients. In that regard, the FRAX™ score appears to be better suited for the purpose of surveying for osteoporotic fall-prone patients.

Administration of anticoagulant and antiplatelet drugs worsened the risk of ICH, as shown by univariate analysis and multiple logistic analyses. In our study, fallers who were given any kinds of anticoagulants and/or antiplatelets suffered ICH six-times more frequently than did fallers who were not given any of these drugs. It is well known that administration of these drugs can increase the risk of ICH in patients (Mattle et al, 1989; Mina et al, 2002 & 2003; Pieracci et al, 2007). Among the various drugs administered to the patients, warfarin was the only drug that was significantly associated with ICH after falls in both the chi-square test and logrank test. However, the effect of warfarin anticoagulation in patients with head trauma is still controversial (Gangavati et al, 2009).

There are several limitations of our study. First, we limited the study subjects to patients aged from 40 to 90 years. This was because the FRAX™ scoring system targets that age group. Second, no validation study using a prospective cohort was performed. Third, this was a retrospective study in a single institute. It is necessary to conduct studies in other acute-care hospitals to ascertain whether reproducible results can be obtained. Finally, and most importantly, the sample size of our study was small in terms of the patients with ICH after falls. Because of the small sample size, we could not divide the study subjects into a development dataset and a test dataset nor construct a proper statistical model. Further investigation using a larger sample size is needed.
